# Michaela Polacek. ‘I had to eat myself up on the left, now I’m only on the right’

**DOI:** 10.1017/S2045796024000714

**Published:** 2024-11-27

**Authors:** Florian Reese

**Affiliations:** ATELIER 10, Vienna, Austria

Michaela Polacek was only interested in art in a very superficial way until she was twenty-eight, or so it seemed. She had many plans for her secure, middle-class life – an artistic career was certainly not one of them.

Today, looking back 22 years later, it is even more astonishing with what urgency, passion, and above all immediacy she turned to this profession. An unexpected rupture in her life was undoubtedly the trigger for the abrupt paradigm shift. But it was her persistence, especially as a newcomer, and her deeply rooted talent that provided the high-energy and unorthodox dynamic that we attribute to the phenomenon of art.

From the very beginning a development could be observed in Michaela Polacek that is often fitted into the art-historically separated schematic catalogue of so-called Outsider Art or Art Brut.

Michaela Polacek was born in 1972 in the Austrian capital of Vienna. She grew up in a well-to-do environment and worked as an office employee after a commercial apprenticeship. Five years later Polacek quit her job. To counteract the work structure that she had previously considered too stereotypical, she used the time off to look for stimuli and alternatives. She worked in the catering industry and obtained a business license. She later got a motor yacht and pilot’s license.

Shortly after attempting to return to work, however, she fell ill. As of 1999, completely unexpectedly, Polacek had to undergo psychiatric treatment for a long period. This situation set her back socially and rattled her self-image. At least at the beginning of this turning point.

In hospital, she was advised to use the usual arts and crafts media aimed at keeping her occupied and calming her down. However, Polacek did not bother long with the offer of decorative or therapeutic output. Instead, the boredom of her hospitalisation brought out free, previously unimagined artistic impulses. Polacek found her original artistic resources, her own techniques, and, it seems, her already slumbering, clear, formal-aesthetic notions of design unusually quickly.

In 2002, meanwhile discharged from hospital, Michaela Polacek found out about Atelier Gugging, an open studio about 20 kilometres from Vienna that offers free artistic activity primarily for people with disabilities and illnesses. Atelier Gugging was founded in 2001 as part of the expanded program of the Art Brut Center Gugging. In the following years, Polacek regularly utilized the studio’s offer. Her extraordinary talent was rapidly noticed here, although she initially only used small paper formats. Unlike today, she still worked with narrative subjects. Her absurd-comic illustrations, which always centred on eccentric figures and characters, were technically unpretentious and free of school conventions. Nevertheless, they exuded a peculiar charm, inventiveness and drawing finesse. She kept developing new series, working with fine liners, ink and pen, watercolours, and occasionally with acrylic paints.

**Figure 1.**
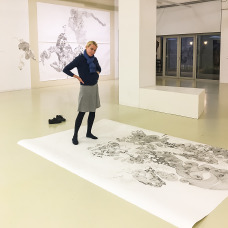
Michaela Polacek in the gallery of Atelier 10, 2018.

But the local setting, the relatively small space of the shared studio, and the only dim prospects of using the results of her work to reinvent herself in her new role as an artist, to gain attention, and ultimately to be successful professionally, hindered her further development. Polacek therefore withdrew for a while and only worked sporadically in her apartment.

When Atelier 10, a new art platform, opened in Vienna in 2012, Michaela Polacek immediately applied. She found sufficient space, peace, and the right level of specialised structure for her. Within the first six months, Polacek was highly motivated to experiment with different drawing materials, themes, and subjects – but above all she strove for ever larger formats. For her participation in the first Atelier 10 exhibition in September of the same year,^1^ she created one of her first larger drawings. After having rejected numerous attempts as not being good enough, she managed to create a drawing on a 198 × 132-centimetre-large paper format using pencil, fine liner and ink pen that was groundbreaking in its dimensions, technical properties, and style.

**Figure 2.**
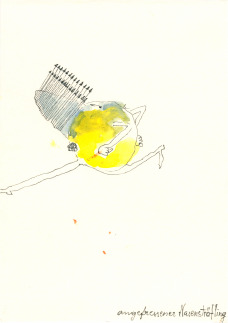
‘Angefressener Nasensträfling’ (annoyed nose-convict), Michaela Polacek, 2009, inkpen and water colour on paper, 14,8x10,4 cm.

Polacek’s work is characterised by great intensity to this day. She produces works that are as complex as they are immediately emotional. They are largely non-representational, but are rooted in hidden figures, with Brustbusenmadonnen (breast bosom madonnas) or women’s heads, whose grimaces, hairstyles and coverings are important reference points for her, a kind of guide and basic order in the composition of the picture. While her earlier works were more reserved and illustrative, today she combines figures and objects, body parts, architectural elements, and ornamentation into free graphic chains of association, into elaborate compositions of sometimes baroque density and opulence. Her motifs at times extend to paper surfaces of up to 200 × 300 centimetres. The structure of her creations is sometimes reminiscent of cartography, thickets of plants, martial gadgetry or internal organic worms. Sometimes her drawings appear like images of microorganisms, sometimes they seem like something very large, comprehensive and macrocosmic.

Polacek wants her design to remain cryptic and rationally inaccessible, like a wild dream. Analysing her works iconographically to synthesise them again later makes no sense from an artistic point of view, as this process would not optimise the comprehensibility of her art as such. Polacek is therefore not interested in the objectivity of the individual image elements and an objective interpretation of her work. Everything is designed for effect, for emotionalisation.

In fact, Michaela Polacek manages to concentrate on the essentials artistically despite the excessive graphics and image elements. She plans her works of art only far enough in advance to still have enough scope for creative outbursts. She only creates her sketches, preliminary constructions and graphic filler spaces to be able to question, circumvent or diligently ignore them in the course of the work process. She rigorously discards pencil sketches that have already been applied, which in the end only remain on the paper like shadows of initial thoughts. Occasionally she leaves large parts of the sheet blank, occasionally her drawing clouds push into nothingness, beyond the edge of the picture.

**Figure 3.**
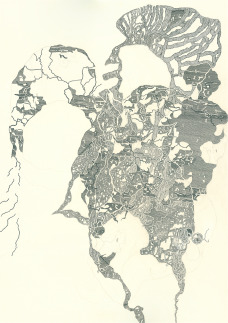
Archaischer Mensch u. 3-Ohrenspitzmaus auf Henkeldingsbums’ (archaic human and 3-ears-shrew on handle-thingamabob), Michaela Polacek, 2017, inkpen and pencil on paper, 100x70 cm, Collection Hannah Rieger.

Michaela Polacek is aware that her minutely detailed technique, her impressive craftsmanship, cannot be the only thing that should be the focus when it comes to assessing her artwork. Therefore, with each new picture she produces, she wages, as she says, a real battle against threatening artistic favours and mundane, decorative harmonies. She was helped not only by the artistically demanding environment, the exchange and comparison with curators and artists in the studio setting, but also by her burgeoning interest in other artists, including those from the so-called Art Brut circle, whom she met at exhibitions in Gugging. Polacek is fascinated by the art of Guo Fengyi, Martín Ramírez, Adolf Wölfli, Anna Zemánková, Unica Zürn and especially Marta Grunenwald. By no means is Polacek attracted by the socially romantic aura of the outcasts that is often projected onto these artists. Rather, like most artists, she is looking for constant positioning, new design parameters and, of course, eclectic ideas. In the oeuvre of the artists mentioned, who work very differently, she also seeks confirmation of her own premises, for dissonances, inconsistencies and the lack of slickness in the art of drawing.

Many years ago, Polacek wrote on a piece of paper, seemingly incidentally: ‘I had to eat myself up on the left, now I’m only on the right’. This laconic statement is, on the one hand, driven by a diffuse feeling of pain and loss. On the other hand, it is astonishing because it works with the euphony of lyrical densification. Polacek’s interest in the coded is reflected in the titles she gives her drawings. In her early illustrative works, the titles still play an important, narrative-accompanying role – even if they are only partially conducive to the objective understanding of the picture story. They function like sidekicks that add an additional pleasant touch to the confusion. They are titles such as ‘The Vampire grey-pigeon red-toothed-grapehood’, ‘The pilchard-pilchardistic anchovy-tail-diner’, ‘The finger-ban devour-convict’, ‘The skyscraper-hat snotty-and-antisnotty-nose-empress’ or ‘The blue-phew-poodle snoot-tooth-breast chocolate-vanilla-pudding-headscarf sniffing-trunk shoe-toast-casserole-sandwich-loner’. She retained the virtuosity of her captions, some of which are neologisms, but above all serpentine and coupling words, in her later large-format works, and at the same time relieves her even more of the supposed task of explaining the work of art: ‘2 missencounter-people and a looking-man behind/between handle-lemon’ or ‘headed and hairy chignon-hair U-hooked and watered’.

The more Polacek identified at first with the role of the artist, the more she had to realise that, in contrast to other fields of work, the art world is an area hardly objective in terms of quality and riddled with erratic rules. Polacek therefore looked early on for professional affiliations and relationships. Due to her psychiatric illness, her non-academic, autodidactic career, and her time at Gugging, her art was quickly placed close to the categories of Art Brut and Outsider Art – categories that she initially rejected in relation to her own work, however, because she feared that, in addition to her depressing social outsider role, her art would also by definition occupy a marginal niche.

As a matter of fact, these categories play a special role in art history and are, therefore, from today’s perspective, not unproblematic. The art summarised under these headings is unusually heterogeneous regarding formal aesthetics. In a departure from convention, they do not describe any stylistic similarities or a concise technique.

Both categories emerged many decades ago, during times of social and art-historical upheaval. The French artist and collector Jean Dubuffet invented the term Art Brut in 1947, that is, at a time of artistic and social new beginnings. Throughout his life, Dubuffet claimed the term exclusively for his collection of art by psychiatric patients, hermits, and other outsiders. The 1970s were also marked by profound cultural ruptures. In this atmosphere, the British art critic Roger Cardinal wrote a book in 1972 about the art of ‘hermits, madmen, dropouts, innocents and spiritual fanatics’ entitled Outsider Art.^2^ If one understands the terms through their historical context of validity in the 1940s and 1970s, one must question their relevance today. The terms Art Brut and Outsider Art are therefore not intended to temporally limit or restrict a specific art-historical movement.

A particular explosive force arises, however, from the social exclusivity that surrounds these categories. The actual common denominator is not defined by the appearance of the art, but by the psychosocial state of the artists, by their social marginal position: a large proportion of these artists are mentally or cognitively impaired. There would be nothing wrong with this special path in art history, provided that the artists concerned participate in the discourse themselves and agree to be classified as an artistic marginal position. In practise, however, this only happens very rarely. This gives the impression that Art Brut or Outsider Art is primarily art for and by curators. The sovereignty of interpretation lies with them. A common notion inherent in art is defined solely by theorists, not the artists themselves.

The large psychosocial gap between artists and their mentors is often reflected in public relations work. It often seems as if a para-artistic track is being provided for social outsiders. Jean Dubuffet divided the art world into two separate orders: that of the arts culturels, ‘the usual art (…) the polished one’ and that of ‘Art Brut, art in its raw form’. Dubuffet strictly rejected a merging of the two orders.^3^ To date there are only a few experts who clearly advocate locating art and artists at the centre of cultural society and at the same time take the appropriate measures. Exotic features can still be found in exhibitions and subtexts that fuel the appeal of the pathological and celebrate the authenticity of the naive. Moreover, adult artists with cognitive disabilities are frequently written about in childish and trivialising language, contradicting any equality.

With so much exclusivity, the question must be allowed as to the extent to which the call for the inclusion of outsiders in the general art canon can be formulated as a credible goal. Is full participation being sought, or is this discourse more of a mode of struggle that suffices, pleases and sustains itself?

In order to re-discuss this ethical component of this paradox after decades of stagnation, the curators of Atelier 10 regularly exchange ideas with representatives and supporters of these categories, since both sides want to see artists destigmatised. Atelier 10 advocates using labels such as Art Brut or Outsider Art solely when they are reflected upon and expressly authorised by the artists themselves.

And Michaela Polacek? Whether her drawings fit into the canon of traditional Art Brut or Outsider Art is irrelevant to her today. Polacek now readily accepts being classified as an Art Brut artist – as long as it does not relativise her achievements and stand in the way of her recognition as a contemporary artist.

## Footnotes

1 Veränderung an allgemeinen Teilen, 20 September 2012–28 March 2013.

2 blurb, Praeger Publishers, New York, Washington.

3 *Malerei in der Falle: Antikulturelle Positionen*, Schriften Band I, ed. Andreas Franzke [Bern-Berlin: Verlag Gachnang & Springer, 1991], p. 82).

